# Predicting the potential suitable distribution area of *Emeia pseudosauteri* in Zhejiang Province based on the MaxEnt model

**DOI:** 10.1038/s41598-023-29009-w

**Published:** 2023-01-31

**Authors:** Sheng Li, Zesheng Wang, Zhixin Zhu, Yizhou Tao, Jie Xiang

**Affiliations:** 1 College of Landscape Architecture, Zhejiang A&F University, Hangzhou, 311300 China; 2 China Celadon College, Lishui University, Lishui, 323000 China; 3 Zhejiang A&F University Landscape Design Institute Co., Ltd., Hangzhou, 311300 China

**Keywords:** Biodiversity, Ecological modelling, Biodiversity, Conservation biology, Ecological modelling

## Abstract

Human activities, including urbanization, industrialization, agricultural pollution, and land use, have contributed to the increased fragmentation of natural habitats and decreased biodiversity in Zhejiang Province as a result of socioeconomic development. Numerous studies have demonstrated that the protection of ecologically significant species can play a crucial role in restoring biodiversity. *Emeia pseudosauteri* is regarded as an excellent environmental indicator, umbrella and flagship species because of its unique ecological attributes and strong public appeal. Assessing and predicting the potential suitable distribution area of this species in Zhejiang Province can help in the widespread conservation of biodiversity. We used the MaxEnt ecological niche model to evaluate the habitat suitability of *E. pseudosauteri* in Zhejiang Province to understand the potential distribution pattern and environmental characteristics of suitable habitats for this species, and used the AUC (area under the receiver operating characteristic curve) and TSS (true skill statistics) to evaluate the model performance. The results showed that the mean AUC value was 0.985, the standard deviation was 0.011, the TSS average value was 0.81, and the model prediction results were excellent. Among the 11 environmental variables used for modeling, temperature seasonality (Bio_4), altitude (Alt) and distance to rivers (Riv_dis) were the key variables affecting the distribution area of *E. pseudosauteri*, with contributions of 33.5%, 30% and 15.9%, respectively. Its main suitable distribution area is in southern Zhejiang Province and near rivers, at an altitude of 50–300 m, with a seasonal variation in temperature of 7.7–8 °C. Examples include the Ou River, Nanxi River, Wuxi River, and their tributary watersheds. This study can provide a theoretical basis for determining the scope of *E. pseudosauteri* habitat protection, population restoration, resource management and industrial development in local areas.

## Introduction

Zhejiang Province has experienced rapid economic growth since the 1970s, but factors such as urbanization, industrialization, modern agricultural pollution, and changes in the way land is used have had significant negative impacts on ecological environments and patterns of landscape security; as a result, there has been gradual fragmentation of natural habitats as well as declines in biodiversity^1,2^. Additionally, some ecologically vulnerable animals in nature can serve as warnings to humans through changes in population size and habitat range^3^. For survival, fireflies (Coleoptera: Fluoridae) need habitats that are ecologically stable and diversified and meet their demanding standards of ecological quality. Since they can periodically reflect local ecological changes and provide early warning of natural environmental changes, fireflies are usually regarded as excellent environmental indicator species^4,5^. In addition, fireflies are regarded as one of the most beautiful insects in the world because of how they use their distinctive bioluminescence for courting and communication^6,7^. Currently, Zhejiang Province is home to approximately 10 different species of fireflies, with *Pyrocoelia* and *Luciola* being the most common genera. It is concerning that large firefly populations are now uncommon and occur only in a handful of locations and that certain confined, dispersed, and fragmented habitats could vanish at any moment as a result of commercial development, artificial light pollution, and the threat of pesticides. According to pertinent research investigations, habitat loss has emerged as the greatest threat to fireflies globally^8^. In Zhejiang Province, both the frequency of occurrence and the size of the firefly population have significantly decreased in recent decades^9^. The loss of firefly habitat and the population decline have also, to some extent, induced changes in the populations of birds, fish, insects, and other creatures, compounding the decline in biodiversity. Fireflies are a crucial link in the food chain of the ecosystem. The restoration of biodiversity, however, can be greatly aided by the protection of species with specific ecological importance, according to research^10^. Research on the preservation of firefly populations and habitats in Zhejiang is needed, given the significance of fireflies in relation to biodiversity and environmental stability in Zhejiang Province.

From the perspective of biodiversity conservation practices, understanding the spatial distribution patterns of species is essential for species conservation^11^. Species distribution models (SDMs) are important for species habitat management because they can use existing species distribution information and relevant environmental data to predict suitable distribution areas and assess and filter key environmental factors^12^. Several SDMs have been successfully developed and implemented, including MaxEnt (maximum entropy model)^13^, ENFA (ecological niche factor analysis)^14^, GLM (generalized linear model)^15^, GAM (generalized additive model)^16^, GARP (genetic algorithm for rule-set prediction)^17^, BIOCLIM^18^ and CLIMEX^19^. Among them, the MaxEnt model is based on machine learning and maximum entropy theory, using known species distribution records and their associated environmental variables to infer the ecological demand of species and then predict the potential distribution range of species in the study area. Compared with other models, the MaxEnt prediction results are more accurate and stable and have gradually become one of the most widely used SDMs^13,20,21^. In recent years, many scholars have used MaxEnt to predict and assess the suitable distribution areas of insect species similar to fireflies, and the final model prediction results are better and can play a guiding role in understanding the potential distribution of insect species and formulating habitat conservation measures^22–24^.

*Emeia pseudosauteri* belongs to the genus *Emeia* of the family Lampyridae (Coleoptera), a new species of firefly discovered in 2012 in Emei Mountain, Leshan, Sichuan Province^25^. According to the survey, a large-scale natural colony of *E. pseudosauteri* has been occurring annually in Kowloon National Wetland Park, Lishui city, Zhejiang Province, since 2014, and its population size and habitat range have been increasing year by year. The annual firefly glow show staged in the local area is as bright and spectacular as a night sky covered with stars, and this light can induce a strong visual impact and emotional response in viewers, attracting a large number of tourists, gradually forming an ecotourism project in the local area with the frefly viewing experience as the main focus, and bringing tangible economic benefits to the people^26^. However, the excessive interference of human activities, domestic sewage, pesticides, night lights, road hardening, and the proliferation of tourists are causing damage and pollution to the existing habitat of *E. pseudosauteri* in Zhejiang Province, making the survival and reproduction of the species in crisis^27–29^. Similar to other species of fireflies, *E. pseudosauteri* requires stable and high-quality habitats for eggs, larvae and adults and is highly sensitive to environmental changes and disturbances by human activities^25^, making it an excellent environmental indicator species. In addition, it requires habitat conditions that cover the habitat needs of other similar species and fill a predator niche in a typical environment, and thus, it acts as an umbrella species to maintain the balance and stability of the ecosystem^30,31^. More importantly, *E. pseudosauteri* fledge as adults in March–April each year, earlier than other species of fireflies, and the high population densities make it easy to create large areas of stunning firefly glitter^25^. These advantages have made it a local ecotourism icon in just a few years, and it can serve as a promotional and representative flagship species to raise public awareness of ecological conservation^32,33^. Therefore, as a highly valuable insect resource, research on the conservation and utilization of *E. pseudosauteri* must be given full attention. In this paper, we used the MaxEnt ecological niche model to study the distribution status and potential suitable distribution areas of *E. pseudosauteri* in Zhejiang Province, analyze the key environmental factors affecting the natural distribution of this species, and provide a scientific basis for future habitat conservation, population rebreeding, protected area management and industrial development.

## Overview of the study area

Zhejiang Province is located in the Yangtze River Delta region on the southeast coast of China, with geographical coordinates of 118° 01ʹ–123° 10ʹ E; 27° 06ʹ–31° 11ʹ N. The straight-line distance from east to west and from north to south in Zhejiang is approximately 450 km, and the land area is approximately 105,500 km^2^, with the topography sloping from southwest to northeast. The topography is complex, with mountains accounting for 74.6%, surface water accounting for 5.1%, and flat land accounting for 20.3%. Zhejiang Province has a humid subtropical monsoon climate with significant monsoons, four distinct seasons, abundant light, abundant rainfall and humid air. The annual precipitation ranges from 1100 to 2000 mm, and the average annual temperature ranges from 15 to 18 °C^34^. The forest vegetation in Zhejiang Province is a central subtropical evergreen broad-leaved forest, and the main forest vegetation types are warm coniferous forest, evergreen broad-leaved forest, deciduous broad-leaved forest, mixed evergreen deciduous broad-leaved forest, mixed coniferous broad-leaved forest, bamboo forest, economic forest, and shrub forest^35^.

## Materials and methods

### Distribution data for ***E. pseudosauteri***

**Figure 1:**
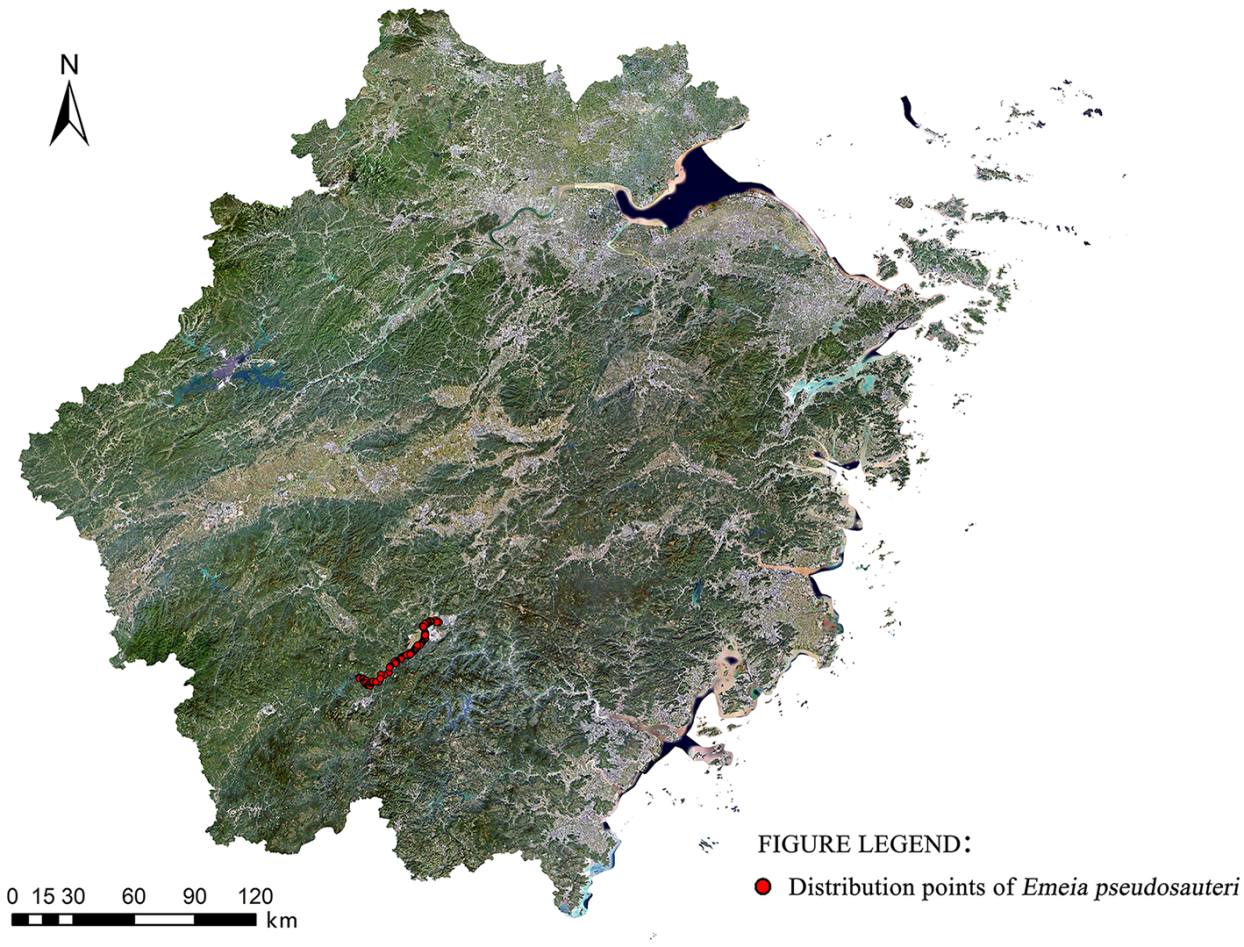
Map of distribution data of *E. pseudosauteri* in Zhejiang Province as of April 2022 (Bigemap v25.5.0.1: http://www.bigemap.com/index.html).

From 2014 to the present, our research team has been continuously investigating and recording the distribution data of *E. pseudosauteri* in Lishui city, Zhejiang Province. Based on the observation results over the years, its distribution range has been gradually expanding. From 2014 to 2015, the distribution of *E. pseudosauteri* in Kowloon National Wetland Park, expanded from 0.17 to 4 km^2^; from 2016 to 2017, it was found in approximately 80% of the park, covering an area of 13.5 km^2^. Starting in 2018, its habitat gradually expanded upstream and downstream along the Daxi Creek watershed, where Kowloon National Wetland Park is located, with a total of three larger aggregations occurring. As of April 2022, we obtained a total of 282 *E. pseudosauteri* distribution sites through field research (Fig. 1). Artificial sampling may bias sampling toward easily accessible areas, and because of the significant spatial clustering of small habitat distribution points, model predictions tend to be overfitted around known occurrences, and model performance values tend to be inflated^36^. To remove spatial autocorrelation and sampling bias, the obtained distribution data were subjected to 1 km spatial dilution using SDMtoolbox^37^. The final 36-point information was obtained and imported into an Excel sheet and collated and saved as a CSV format file for MaxEnt modeling analysis.

### Data on environmental variables

Based on *E. pseudosauteri* habitat research and related studies, four types of environmental variables were selected: biological environmental (normalized difference vegetation index, distance to rivers), bioclimatic (19 bioclimatic variables including annual mean temperature and isothermality), topographic (elevation, slope, slope direction), and anthropogenic disturbance variables (lighting, land use, distance to roads, distance to settlements). Topographic variables, the normalized difference vegetation index (NDVI), and land use data were obtained from the website of the Resource and Environmental Science Data Center of the Chinese Academy of Sciences (https://www.resdc.cn/Default.aspx). Bioclimatic variables were obtained from the World Meteorological Data website (http://www.worldclim.org/) by downloading a total of 19^38^. Light data were obtained from the Earth Observation Group (EOG) website (https://eogdata.mines.edu/products/vnl/) for the 2021 annual VNL V2 data^39^. River, road, and settlement data were obtained from the National Basic Geographic Information Database 2021 1:1 million geographic vector data set, and Euclidean distance calculations were performed through ArcGIS 10.6 to obtain the distance to river, distance to road, and distance to settlement raster data, respectively. Then, all 28 environmental variable raster data were resampled to a 1 km resolution by ArcGIS 10.6 to ensure the same range boundary, unified geographic coordinates as WGS_1984, and projection coordinate system as WGS_1984_UTM_Zone_50N. Finally, the processed environmental variable raster data were converted to ASCII format.

Spatial autocorrelation between environmental variables may affect the analysis of the relationship between species distribution and the environment^40^. To reduce the spatial autocorrelation between environmental variables and avoid the influence of highly correlated environmental variables on model prediction results. The first 19 bioclimatic variables were subjected to principal component analysis, and the top three with the highest contribution rates were selected as principal components based on the accumulation of eigenvalues. The standardized eigenvectors of each variable were obtained (Table 1), and then Pearson correlation analysis was conducted on 28 environmental variables (Supplementary Table S1). If the correlation coefficient |r|≥ 0.8, the two variables were considered to be highly correlated, and only the environmental variables had larger eigenvector values^41^. Eleven environmental variables, including elevation, slope direction, slope, seasonal variation in temperature, annual precipitation, lighting, land use, NDVI, distance to roads, distance to rivers, and distance to residential sites, were finally screened for *E. pseudosauteri* species distribution prediction modeling (Table 2).

**Table 1:** Principal component analysis results of 19 bioclimatic variables.

Variables	Eigenvectors		
	PC1	PC2	PC3
Annual mean temperature (Bio1)	− 0.0022	0.0024	− 0.0284
Mean diurnal range (Bio2)	0.0006	0.0077	0.0012
Isothermality (Bio3)	0.0044	0.0032	− 0.0040
Temperature seasonality (Bio4)	− 0.1385	0.6772	0.3058
Max. temperature of the warmest month (Bio5)	− 0.0036	0.0157	− 0.0236
Min. temperature of the coldest month (Bio6)	− 0.0011	− 0.0109	− 0.0346
Temperature of annual range (Bio7)	− 0.0025	0.0265	0.0111
Mean temperature of the wettest quarter (Bio8)	− 0.0062	0.0094	0.0227
Mean temperature of the driest quarter (Bio9)	− 0.0032	− 0.0009	− 0.0212
Mean temperature of the warmest quarter (Bio10)	− 0.0040	0.0103	− 0.0242
Mean temperature of the coldest quarter (Bio11)	− 0.0007	− 0.0074	− 0.0328
Annual precipitation (Bio12)	0.8275	− 0.2352	− 0.1132
Precipitation of the wettest month (Bio13)	0.1704	0.2482	− 0.0910
Precipitation of the driest month (Bio14)	0.0008	− 0.0221	− 0.0215
Precipitation seasonality (Bio15)	0.0116	0.0378	0.0115
Precipitation of the wettest quarter (Bio16)	0.4209	0.6242	− 0.2064
Precipitation of the driest quarter (Bio17)	0.0411	− 0.0756	− 0.1482
Precipitation of the warmest quarter (Bio18)	0.2902	− 0.0766	0.8817
Precipitation of the coldest quarter (Bio19)	0.0604	0.1410	− 0.1936
Contribution rate	94.60%	3.20%	1.30%
Cumulative contribution rate	94.60%	97.80%	99.11%

**Table 2:** Environmental variables used in predictive modeling of *Emeia pseudosauteri* species distribution.

Variable abb	Description	Source
Bio_4	Temperature seasonality	WorldClim database Version 2.1
Bio_12	Annual precipitation	
Light	Lighting	Earth observation group
LUCC	Land use	Institute of Geographical Sciences and Natural Resources Research, Chinese Academy of Sciences
NDVI	Normalized difference vegetation index	
Asp	Slope direction	
Slope	Slope	
Alt	Altitude	
Riv_dis	Distance to river	National basic geographic information database
Res_dis	Distance to settlement	
Roa_dis	Distance to road	

### Maxent model selection and construction

After the *E. pseudosauteri* distribution data and environmental variable data were processed, modeling operations were performed using MaxEnt (version 3.4.4). Twenty-five percent of the distribution points were randomly selected as the test data set, and the remaining 75% of the distribution points were used as the training data set. The maximum number of iterations of the model was set to 500, the number of repetitions was set to 10, the category of repetition was selected as cross-validation, and the relative contribution of 11 environmental variables to the prediction of the suitable distribution area of *E. pseudosauteri* was calculated using the knife cut analysis of the software. The threshold value of each environmental factor was responded to by the response curve, other parameters were set by default, and the prediction results were output. ASCII format was chosen for the format^42^.

## Results and analysis

### Evaluation of model prediction results

AUC (area under the receiving operator curve) is a statistic widely used to evaluate the prediction performance of species distribution models^43,44^, but related studies have proven that AUC is insufficient as a predictor in spatial distribution modeling^45^. Therefore, we introduce TSS (true skill statistics) as a supplement, which inherits all the advantages of Kappa statistics and has a significant correlation with AUC statistics^46^. We refer to the study by Liu et al.^47^, select maxSSS (maximum sum of sensitivity and specificity) as the threshold, and calculate the TSS average of the 10 repeated operation results of the MaxEnt model to evaluate the model performance. The AUC value is generally between 0.5 and 1, and the higher the value is, the better the prediction effect of the model^48^. When the AUC is less than 0.7, it indicates poor model performance, 0.7–0.9 indicates moderate model performance, and 0.9–1.0 indicates good model performance^49^. In the TSS value range, 0.2–0.5 is poor, 0.6–0.8 is useful, and greater than 0.8 is excellent^50^.

**Figure 2:**
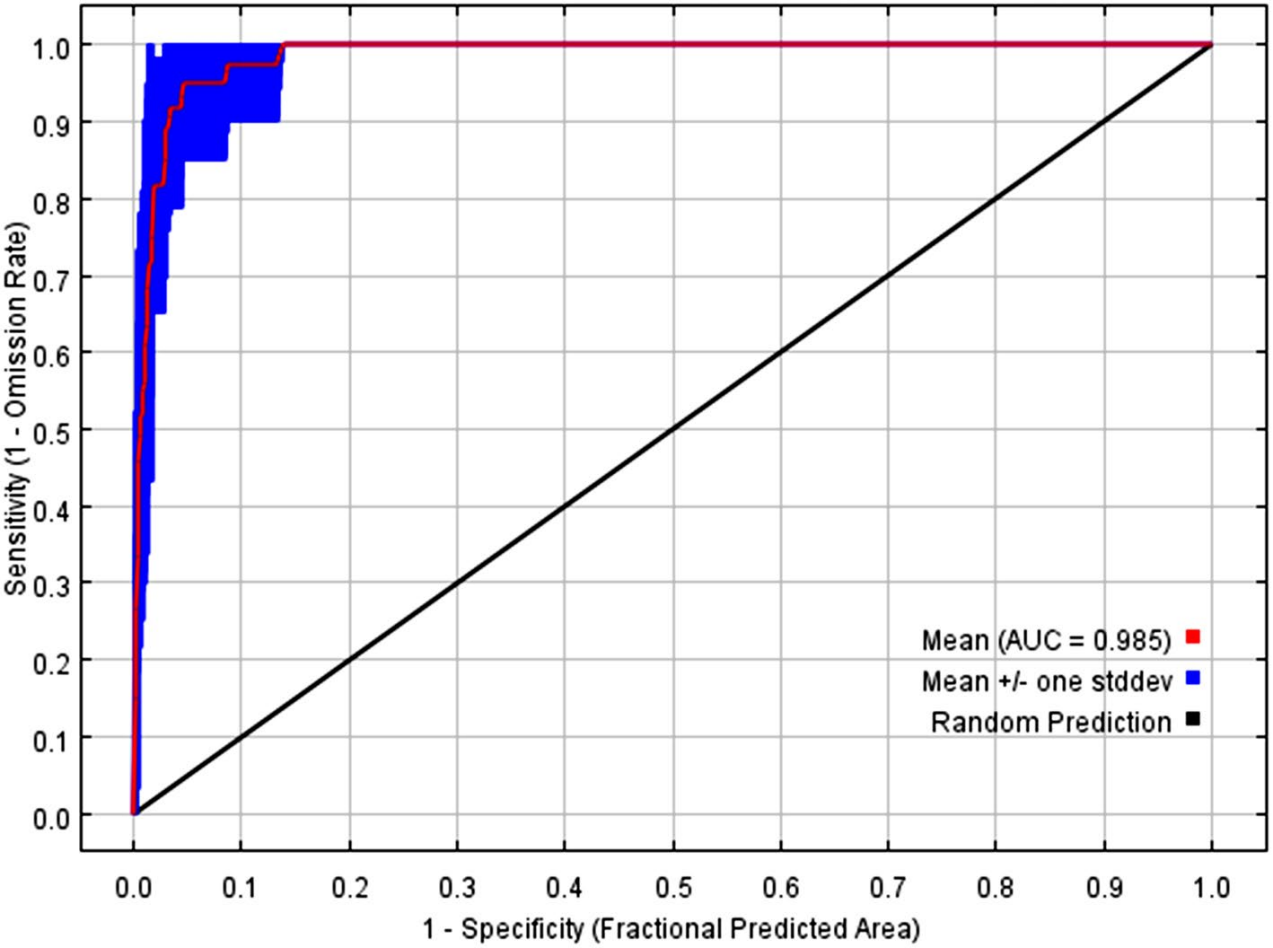
ROC curves and AUC averages for MaxEnt with 10 repeated runs.

The mean AUC of 10 repeated runs of the final model was 0.985, the standard deviation was 0.011 (Fig. 2), and the mean TSS was 0.81 (Table 3). It shows that the model has good performance and high prediction accuracy. The species distribution data and environmental variable data used in the modeling can effectively predict the potential suitable distribution area of *E. pseudosauteri* in Zhejiang Province.

**Table 3:** Average TSS of the results of 10 iterations of the MaxEnt model.

Models	Threshold	TSS
*Emeia_sp_0*	0.33	0.73
*Emeia_sp_1*	0.26	0.72
*Emeia_sp_2*	0.33	0.48
*Emeia_sp_3*	0.13	0.95
*Emeia_sp_4*	0.3	0.73
*Emeia_sp_5*	0.31	0.98
*Emeia_sp_6*	0.27	0.64
*Emeia_sp_7*	0.21	0.97
*Emeia_sp_8*	0.12	0.94
*Emeia_sp_9*	0.3	0.98
		Mean = 0.81

### Contribution of environmental variables

**Figure 3:**
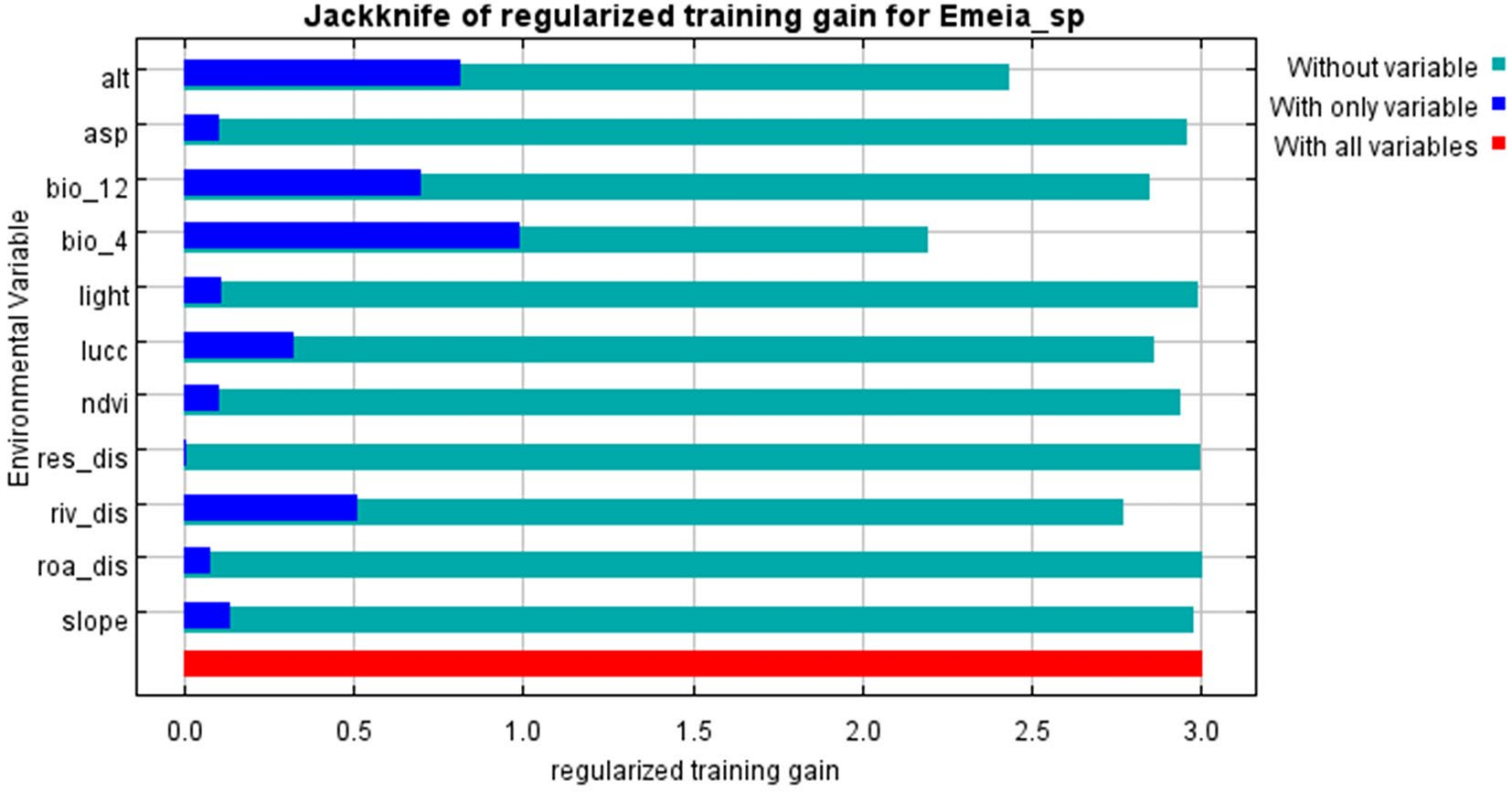
The importance of jackknife analysis of environmental variables.

We analyzed the importance and contribution of each environmental variable in the model prediction by the jackknife method in the MaxEnt prediction model. The results showed that the contributions of bio_4 (temperature seasonal variation) and Alt (altitude) were 33.5% and 30%, respectively, and the regularized training gain values were both greater than 0.75, which can provide a greater benefit to the prediction model. Thus, bio_4 and Alt had more useful information than the other environmental variables and had the greatest effect on the distribution of *E. pseudosauteri* in Zhejiang Province. Additionally, riv_dis (distance to rivers), bio_12 (annual precipitation) and LUCC (land use) were the three environmental variables with the next highest contribution and regularized training gain values and had some influence on its distribution in Zhejiang Province. The remaining environmental variables, including the NDVI, slope direction, slope, light, distance from roads, and distance from settlements, had a small or even negligible effect on its distribution (Table 4, Fig. 3).

**Table 4:** Percent contribution of environmental variables in the MaxEnt model.

Variable abb	Description	Contribution (%)
Bio_4	Temperature seasonality	33.5
Alt	Altitude	30
Riv_dis	Distance to river	15.9
LUCC	Land Use	7.2
Bio_12	Annual precipitation	5.9
NDVI	Normalized difference vegetation index	3.3
Asp	Slope direction	1.9
Slope	Slope	1.1
Light	Lighting	0.8
Res_dis	Distance to settlement	0.2
Roa_dis	Distance to road	0.2

### Response curve analysis of environmental variables

**Figure 4:**
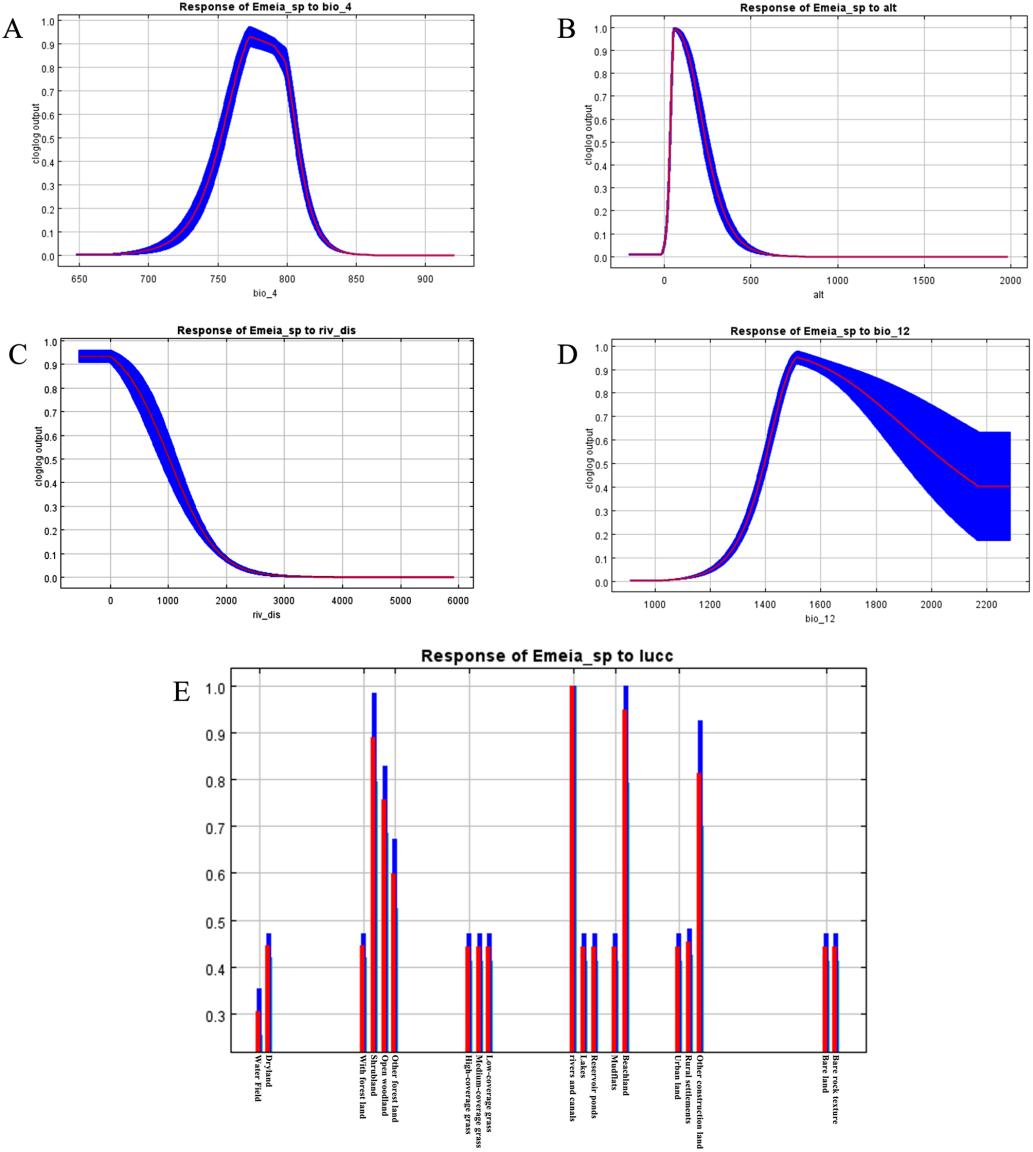
Response curve of the main environmental variables affecting the distribution of *E. pseudosauteri.* (**A**) Response curves of bio_4 (temperature seasonal variation). (**B**) Response curves of Alt (altitude). (**C**) Response curves of riv_dis (distance to rivers). (**D**) Response curves of bio_12 (annual precipitation). (**E**) Response curves of LUCC (land use).

To explain the effect of each environmental variable on the distribution of suitable habitat, the MaxEnt model gives response curves between the probability of species presence and environmental variables^51^. The response curves for the main environmental variables affecting the distribution of *E. pseudosauteri* are shown below (Fig. 4). The seasonal change in the air temperature response curve showed an overall trend of increasing and then decreasing, and its presence probability reached a maximum of 93% at 7.7 °C. When the amount of seasonal change in air temperature was less than 7.7 °C and more than 8 °C, the presence probability decreased rapidly, while the curve changed slowly between 7.7 and 8 °C. Therefore, it can be indicated that it is more sensitive to seasonal temperature changes. In terms of altitude, the presence probability of *E. pseudosauteri* increased rapidly with increasing altitude, reaching an extreme value at 100 m, after which the presence probability gradually decreased with increasing altitude. This result suggests that a specific altitude can provide a unique and suitable habitat for it. The presence probability of *E. pseudosauteri* was negatively correlated with the distance from the river, with the presence probability decreasing as the distance from the river increased. The probability of *E. pseudosauteri* survival was most suitable when the annual precipitation was approximately 1500 mm, and the distribution probability reached an extreme value of 95%. Between 1000 and 1500 mm, the probability of presence gradually increased and then gradually decreased. The response curves of land use variables indicated that *E. pseudosauteri* habitat selection preferred mudflats, river canals, and shrubby woodlands. Collectively, this species is highly dependent on wetland environments near rivers with high annual precipitation, and the dense vegetation of shrubby woodlands can provide it with sheltered, safe, and stable habitat environments.

### Prediction of suitable habitat for *E. pseudosauteri* in Zhejiang Province

**Figure 5:**
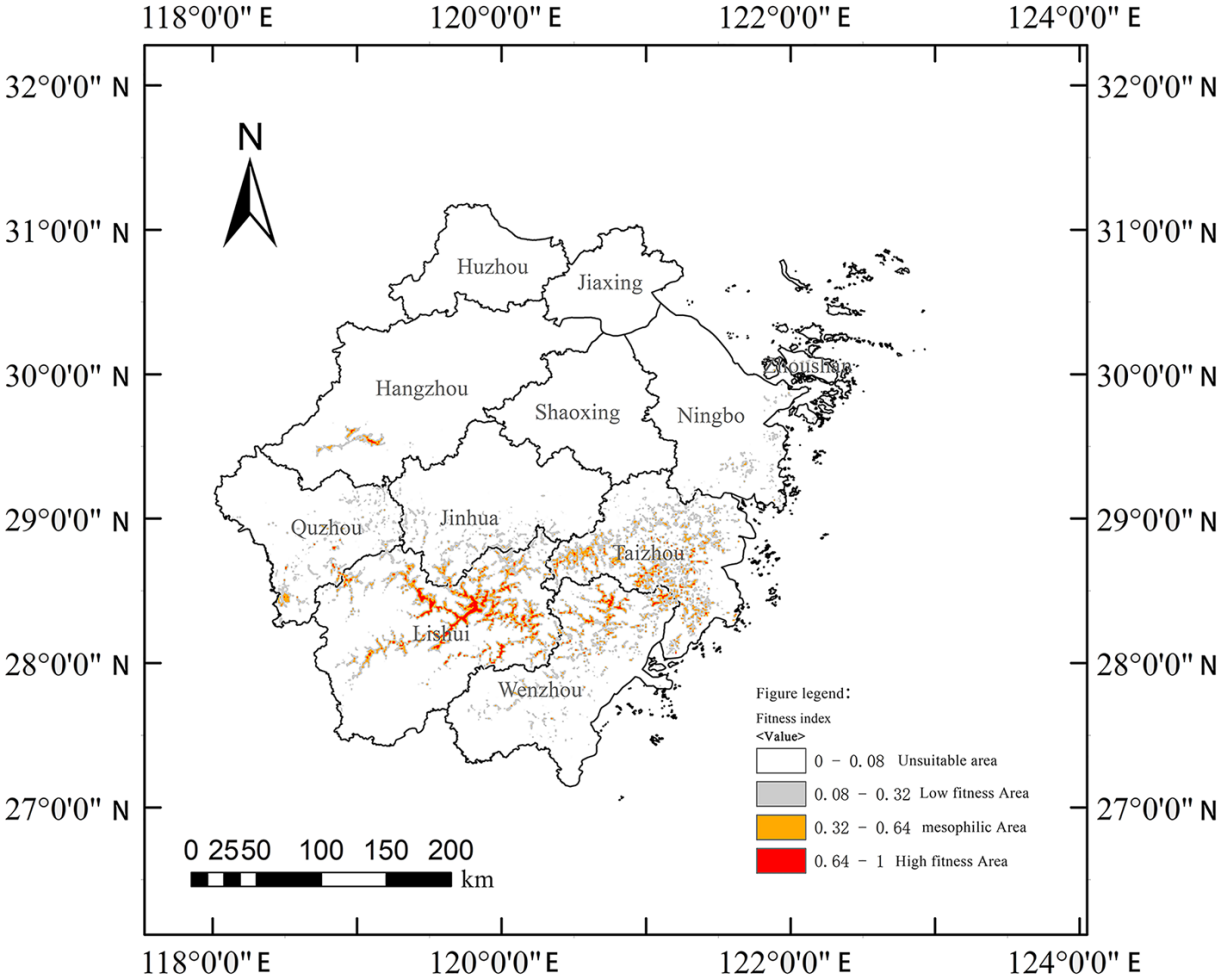
Map of the suitable distribution area of *Emeia pseudosauteri* in Zhejiang Province (MaxEnt v3.4.4: https://biodiversityinformatics.amnh.org/open_source/maxent/, Bigemap v25.5.0.1: http://www.bigemap.com/index.html).

According to the fitness index P output from MaxEnt software, the predicted fitness zones were classified into four levels: 0–0.08, nonsuitable zone; 0.08–0.32, low suitability zone; 0.32–0.64, medium suitability zone; and 0.64–1, high suitability zone. The suitable area for *E. pseudosauteri* was mainly distributed in southern Zhejiang Province, and the highly suitable area was approximately 775.91 km^2^, accounting for approximately 0.74% of the land area of Zhejiang Province; the moderately suitable area was approximately 1458.09 km^2^, accounting for approximately 1.38%; and the less suitable area was approximately 3996.96 km^2^, accounting for approximately 3.79%. The distribution of medium- fitness and high-fitness zones was more scattered, and a continuous larger area of high-fitness zones was found only in Lishui city (Fig. 5).

Highly suitable areas were: Daxi watershed in Lishui city, Longquan Creek and Jingshuitan watershed in Yunhe County, Songyin Creek watershed in Songyang County, parts of Longquan City, Xiaoxi and Qianxia Lake watersheds in Qingtian County and parts of the Oujiang River watershed from Qingtian County to Wenzhou City; Dannan Creek watershed in Yongjia County, Wenzhou; around Xianxia Lake watershed in Hunan Town, Quzhou city; around Qiandao Lake watershed in Chun'an County, Hangzhou city; around Changtan Reservoir watershed in Huangyan District, Taizhou city.

The distribution of the middle fitness zone was basically the same as that of the high fitness zone, mainly extending outward to a certain extent with the high fitness zone at the center, including the area around Xianxia Lake waters in Lishui city, Dongdu Township in Jinyun County, Lanju Township in Longquan city, Xiakou Township in Quzhou city, Baita Township in Xianju County in Taizhou city, around Niutoushan Reservoir in Linhai city, and some areas in Wenling city, in addition to those in the high fitness zone mentioned above.

## Discussion

The suitable distribution areas predicted by the MaxEnt model essentially reflect a selection made by *E. pseudosauteri* for the natural environment in the region. Among the 11 environmental variables, the seasonal variation in temperature, elevation and distance from rivers were the main factors affecting the habitat suitability and distribution probability of the species. Temperature is an important condition for firefly habitat reproduction, and temperature changes can directly affect firefly populations^52^. Combined with the response curves of environmental variables output from the prediction model, when the amount of seasonal variation in temperature was between 7.7 and 8 °C, it can provide an effective accumulation temperature for *E. pseudosauteri* and ensure the hatch rate of eggs. Elevation is considered to be one of the determinants of species diversity distribution patterns and can influence the climatic characteristics of the habitat, the distribution of water systems, and vegetation community types to some extent^53–55^. From the output of the prediction model, *E. pseudosauteri* tended to select the area between 50 and 300 m elevation as their habitat, and the area between that elevation was mostly hilly. In southern Zhejiang, there are continuous, gently undulating low hills, with a mild climate, abundant precipitation, and diverse and distinct vegetation types, which successfully shape the unique and suitable habitat environment. As terrestrial fireflies, *E. pseudosauteri* exhibits extremely high hydrophilicity, and habitat suitability decreases with increasing distance from rivers, decreasing the probability of *E. pseudosauteri* being present. Based on previous studies, this may be due to egg hatching as well as larval food sources^25^. Compared to the results of other studies on firefly habitats, the effects of environmental variables such as the NDVI, lights, distance from roads, and distance from residential sites on *E. pseudosauteri* were not significant in this study, which may be due to the different scales of research and the existence of scale effects. Moreover, because *E. pseudosauteri* has been a new species of firefly in Zhejiang Province in recent years, it is currently distributed only in part of the Daxi watershed in Lishui city, Zhejiang Province, with a small distribution range and population size. Although it has been demonstrated that the MaxEnt model can also achieve good prediction results with less species distribution data, it may have an impact on the prediction assessment results because the input environmental variables are still relatively coarse at the spatial scale^56,57^. Studying the effects of ecology on species has always been an important basis for capturing the habitat requirements and spatial distribution of species. Based on the results of this MaxEnt model prediction, this research team will conduct long-term monitoring of *E. pseudosauteri* in their medium- to high-suitability distribution areas and, in the future, validate the screening of more comprehensive environmental variables to explain the distribution of *E. pseudosauteri* in their suitability areas through habitat requirements, species behavior, and habitat range studies.

In recent years, increasing numbers of scholars in China have been actively conducting research on firefly habitat restoration, ecological conservation and artificial rebreeding^9^. The relevant research results provide the basis for the exploitation of firefly resources as well as industrial development. The development and utilization of *E. pseudosauteri* value resources need to be fully integrated with other industries, e.g., the development of firefly organic agriculture, so that *E. pseudosauteri*, as an excellent environmental indicator species, can be used to monitor the excellent quality of local organic agriculture and enhance the market competitiveness and added value of local agricultural products^9^. The viewing of fireflies can also improve the health of the heart to a certain extent, thus allowing the development of a corresponding recreation industry^58,59^. In addition, the development of firefly science education, firefly cultural exhibitions, and firefly creative products can make *E. pseudosauteri* a local cultural symbol, bring tangible economic benefits to local people, and stimulate public awareness of conservation. Moreover, a portion of the revenue generated by the development of firefly-related industries can be used for conservation research on habitat ecology and population breeding^60^. Only in this way can the value of *E. pseudosauteri* be used rationally and fully to achieve safe and sustainable development.

## Conclusion

Potentially suitable distribution areas for *E. pseudosauteri* are basically along river waters, mainly in the Ou River, Nanxi River and Wuxi River and their tributaries in southern Zhejiang. Although the medium–high suitable distribution area covered a large and extensive area, the overall suitable habitat area was small and fragmented, which led to a poor ecological carrying capacity and resistance of the habitat and was vulnerable to anthropogenic disturbance. Therefore, it is particularly important to develop corresponding conservation mechanisms to strengthen the regulation and protection of *E. pseudosauteri* habitats. By establishing protected areas, ecological corridors and conservation nodes, we can improve the landscape ecological security pattern of suitable habitats for this species, solving the shortcomings of the current narrow and fragmented habitats and further improving habitat suitability. The *E. pseudosauteri* habitats are diversified and contain wetlands, farmlands, bamboo forests and shrublands, which overlap with many species' habitats and have similar ecological niches, so the conservation of *E. pseudosauteri* will, to a certain extent, contribute to the extensive conservation of biodiversity in Zhejiang Province.

Although this paper focuses on the potential suitable distribution areas and environmental factor characteristics of suitable habitats for *E. pseudosauteri* in Zhejiang Province, there are certain similarities in the habitat environments of different species of fireflies, and the predicted evaluation of the habitat suitability of *E. pseudosauteri* can provide a reference for conservation studies of other species of fireflies, which will have wider research significance.

## Supplementary Information

Supplementary Table S1.

## Data Availability

The original contributions presented in this study are included in the article/supplementary material, and further inquiries can be directed to the corresponding author.

## References

[1] Daskalova GN (2020). Landscape-scale forest loss as a catalyst of population and biodiversity change. Science.

[2] Betts MG (2019). Extinction filters mediate the global effects of habitat fragmentation on animals. Science.

[3] Siddig AA, Ellison AM, Ochs A, Villar-Leeman C, Lau MK (2016). How do ecologists select and use indicator species to monitor ecological change? Insights from 14 years of publication in Ecological Indicators. Ecol. Ind..

[4] Thancharoen A (2012). Well managed firefly tourism: A good tool for firefly conservation in Thailand. Lampyrid..

[5] Hwang, Y. T., Moon, J., Lee, W. S., Kim, S. A. & Kim, J. Evaluation of firefly as a tourist attraction and resource using contingent valuation method based on a new environmental paradigm. *J. Qual. Assur. Hosp. Tour*. **21**(3), 320–336 (2019).

[6] Carlson AD, Copeland J (1985). Flash communication in fireflies. Q. Rev. Biol..

[7] Evans TR, Salvatore D, van de Pol M, Musters CJM (2018). Adult firefly abundance is linked to weather during the larval stage in the previous year. Ecol. Entomol..

[8] Lewis SM (2020). A global perspective on firefly extinction threats. Bioscience.

[9] Cao, C. Q., Zhang, Y., Wang, Y. Z. & He, H. Progress in the research, protection, development and utilization of fireflies. *J. Environ. Entomol*.**1–36** (2022).

[10] Myers N, Mittermeier RA, Mittermeier CG, da Fonseca GAB, Kent J (2000). Biodiversity hotspots for conservation priorities. Nature.

[11] Thorn JS, Nijman V, Smith D, Nekaris KAI (2009). Ecological niche modelling as a technique for assessing threats and setting conservation priorities for Asian slow lorises (Primates:Nycticebus). Divers. Distrib..

[12] Elith J, Leathwick JR (2009). Species distribution models: Ecological explanation and prediction across space and time. Annu. Rev. Ecol. Evol. Syst..

[13] Phillips SJ, Anderson RP, Schapire RE (2006). Maximum entropy modeling of species geographic distributions. Ecol. Model..

[14] Hirzel AH, Hausser J, Chessel D, Perrin N (2002). Ecological-Niche Factor Analysis: How to compute habitat-suitability maps without absence data?. Ecology.

[15] Nelder JA, Wedderburn RW (1972). Generalized linear models. J. R. Stat. Soc. Ser. A (General)..

[16] Hastie, T. J. Generalized additive models. *Statistical models in S. Routledge.* 249–307 (2017).

[17] Stockwell DR, Noble IR (1992). Induction of sets of rules from animal distribution data: A robust and informative method of data analysis. Math. Comput. Simul..

[18] Beaumont LJ, Hughes L, Poulsen M (2005). Predicting species distributions: use of climatic parameters in BIOCLIM and its impact on predictions of species’ current and future distributions. Ecol. Model..

[19] Jung JM, Lee WH, Jung S (2016). Insect distribution in response to climate change based on a model: Review of function and use of CLIMEX. Entomol. Res..

[20] Phillips SJ, Dudík M (2008). Modeling of species distributions with Maxent: new extensions and a comprehensive evaluation. Ecography.

[21] Moreno R, Zamora R, Molina JR, Vasquez A, Herrera MÁ (2011). Predictive modeling of microhabitats for endemic birds in South Chilean temperate forests using Maximum entropy (Maxent). Eco. Inform..

[22] Wang Z (2019). Prediction of potential distribution of the invasive Chrysanthemum Lace Bug, Corythucha marmorata in China based on Maxent. J. Environ. Entomol..

[23] Li A (2020). MaxEnt modeling to predict current and future distributions of Batocera lineolata (Coleoptera: Cerambycidae) under climate change in China. Ecoscience.

[24] Sutherland LN, Powell GS, Bybee SM (2021). Validating species distribution models to illuminate coastal fireflies in the South Pacific (Coleoptera: Lampyridae). Sci. Rep..

[25] Fu, X. H., Ballantyne, L. A. & Lambkin, C. Emeia gen. nov., a new genus of Luciolinae fireflies from China (Coleoptera: Lampyridae) with an unusual trilobite-like larva, and a redescription of the genus Curtos Motschulsky. *Zootaxa*. **3403**(1), 1–53 (2012).

[26] Idris NS (2021). The dynamics of landscape changes surrounding a firefly ecotourism area. Glob. Ecol. Conserv..

[27] Santiago-Blay, J. A. *Silent Sparks: The Wondrous World of Fireflies. Life: The Excitement of Biology*. (2016).

[28] Picchi, M. S., Avolio, L., Azzani, L., Brombin, O. & Camerini, G. Fireflies and land use in an urban landscape: the case of Luciola italica L.(Coleoptera: Lampyridae) in the city of Turin. *J. Insect Conserv*. **17**(4), 797–805 (2013).

[29] Pearsons KA, Lower SE, Tooker JF (2021). Toxicity of clothianidin to common Eastern North American fireflies. PeerJ.

[30] Madruga Rios, O. & Hernández Quinta, M. Larval Feeding Habits of the Cuban Endemic FireflyAlecton discoidalisLaporte (Coleoptera: Lampyridae). *Psyche J. Entomol*. **2010**, 1–5 (2010).

[31] Roberge JM, Angelstam PER (2004). Usefulness of the umbrella species concept as a conservation tool. Conserv. Biol..

[32] Bowen-Jones E, Entwistle A (2002). Identifying appropriate flagship species: The importance of culture and local contexts. Oryx.

[33] Walpole, M. J. & Leader-Williams, N. Tourism and flagship species in conservation. Biodivers. Conserv. **11**(3), 543–547 (2002).

[34] Zhejiang Provincial Bureau of Statistics. *Zhejiang physical geography profile*, http://tjj.zj.gov.cn/col/col1525489/index.html (2022).

[35] Zhejiang Provincial Forestry Department. *Announcement of Forest Resources and Their Ecological Function Value in Zhejiang Province*. Zhejiang Daily. 10.38328/n.cnki.nzjrb.2016.002829 (2016).

[36] Boria RA, Olson LE, Goodman SM, Anderson RP (2014). Spatial filtering to reduce sampling bias can improve the performance of ecological niche models. Ecol. Model..

[37] Brown JL (2014). SDM toolbox: A python-based GIS toolkit for landscape genetic, biogeographic and species distribution model analyses. Methods Ecol. Evol..

[38] Fick SE, Hijmans RJ (2017). WorldClim 2: New 1-km spatial resolution climate surfaces for global land areas. Int. J. Climatol..

[39] Elvidge CD, Zhizhin M, Ghosh T, Hsu F-C, Taneja J (2021). Annual time series of global VIIRS nighttime lights derived from monthly averages: 2012 to 2019. Remote Sens..

[40] WAN, J. *et al.* Predicting the potential geographic distribution of Bactrocera bryoniae and Bactrocera neohumeralis (Diptera: Tephritidae) in China using MaxEnt ecological niche modeling. *J. Integr. Agric*. **19**(8), 2072–2082 (2020).

[41] Zhou R (2021). Projecting the potential distribution of glossina morsitans (Diptera: Glossinidae) under climate change using the MaxEnt model. Biology..

[42] Hill MP, Hoffmann AA, McColl SA, Umina PA (2011). Distribution of cryptic blue oat mite species in Australia: current and future climate conditions. Agric. For. Entomol..

[43] Su H, Bista M, Li M (2021). Mapping habitat suitability for Asiatic black bear and red panda in Makalu Barun National Park of Nepal from Maxent and GARP models. Sci. Rep..

[44] Proosdij AJ, Sosef M, Wieringa J, Raes N (2016). Minimum required number of specimen records to develop accurate species distribution models. Ecography.

[45] Lobo JM, Jiménez-Valverde A, Real R (2008). AUC: A misleading measure of the performance of predictive distribution models. Glob. Ecol. Biogeogr..

[46] Allouche O, Tsoar A, Kadmon R (2006). Assessing the accuracy of species distribution models: Prevalence, kappa and the true skill statistic (TSS). J. Appl. Ecol..

[47] Liu C, Newell G, White M (2016). On the selection of thresholds for predicting species occurrence with presence-only data. Ecol. Evol..

[48] Swets JA (1988). Measuring the accuracy of diagnostic systems. Science.

[49] Pearce J, Ferrier S (2000). Evaluating the predictive performance of habitat models developed using logistic regression. Ecol. Model..

[50] Gama M, Crespo D, Dolbeth M, Anastácio PM (2017). Ensemble forecasting of Corbicula fluminea worldwide distribution: projections of the impact of climate change. Aquat. Conserv. Mar. Freshwat. Ecosyst..

[51] Zhao Y, Deng X, Xiang W, Chen L, Ouyang S (2021). Predicting potential suitable habitats of Chinese fir under current and future climatic scenarios based on Maxent model. Eco. Inform..

[52] Evans, T. R., Salvatore, D., van de Pol, M. & Musters, C. J. M. Adult firefly abundance is linked to weather during the larval stage in the previous year. *Ecol. Entomol*. **44**(2), 265–273 (2018).

[53] Chettri B, Bhupathy S, Acharya BK (2010). Distribution pattern of reptiles along an eastern Himalayan elevation gradient India. Acta Oecol..

[54] Brown, J. H. Mammals on mountainsides: elevational patterns of diversity. *Global Ecol. Biogeogr*. **10**(1), 101–109 (2001).

[55] Gairola S, Sharma CM, Ghildiyal SK, Suyal S (2011). Tree species composition and diversity along an altitudinal gradient in moist tropical montane valley slopes of the Garhwal Himalaya India. For. Sci. Technol..

[56] Pearson, R. G., Raxworthy, C. J., Nakamura, M. & Townsend Peterson, A. Predicting species distributions from small numbers of occurrence records: A test case using cryptic geckos in Madagascar. *J. Biogeogr*. **34**(1), 102–117 (2007).

[57] Hernandez PA, Graham CH, Master LL, Albert DL (2006). The effect of sample size and species characteristics on performance of different species distribution modeling methods. Ecography.

[58] Abe N (2004). Kansei estimation on luminescence of Firefly-Kansei information measurement and welfare utilization. J. Japan Soc. Kansei Eng..

[59] Buckley R (2019). Economic value of protected areas via visitor mental health. Nat. Commun..

[60] Lewis SM (2021). Firefly tourism: Advancing a global phenomenon toward a brighter future. Conserv. Sci. Pract..

