# Supplementary material for: Predicting the potential suitable distribution area of Emeia pseudosauteri in Zhejiang Province based on the MaxEnt model
Source: Sci Rep. 2023 Jan 31;13:1806. doi: 10.1038/s41598-023-29009-w (PMC9889780; doi:10.1038/s41598-023-29009-w)
Supplement: Supplementary file 1 — Supplementary Table S1. [file 41598_2023_29009_MOESM1_ESM.pdf]

Supplementary Table S1. Correlation analysis results of 28 initial environmental variables

	Atlitude	slope	Asp	light	NDVI	LUCC	riv_dis	res_dis	roa_dis	bio1	bio2	bio3	bio4	bio5	bio6	bio7	bio8	bio9	bio10	bio11	bio12	bio13	bio14	bio15	bio16	bio17	bio18	bio19
Atlitude	1.00																											
slope	0.57	1.00																										
Asp	0.01	0.03	1.00																									
light	-0.31	-0.29	0.00	1.00																								
NDVI	0.05	0.10	0.00	-0.19	1.00																							
LUCC	-0.08	-0.08	0.00	0.32	-0.14	1.00																						
riv_dis	0.25	0.21	-0.04	-0.18	0.06	-0.06	1.00																					
res_dis	0.14	0.10	-0.03	-0.04	-0.22	0.09	0.16	1.00																				
roa_dis	0.37	0.27	-0.04	-0.19	0.02	-0.05	0.25	0.26	1.00																			
bio1	-0.75	-0.45	0.05	0.29	-0.07	0.09	-0.22	-0.13	-0.30	1.00																		
bio2	0.06	-0.04	0.03	-0.04	-0.08	-0.04	0.09	-0.03	-0.03	0.12	1.00																	
bio3	0.40	0.23	0.02	-0.14	0.00	-0.06	0.13	0.00	0.15	0.00	0.77	1.00																
bio4	-0.59	-0.45	0.01	0.20	-0.13	0.05	-0.09	-0.05	-0.31	0.20	0.16	-0.49	1.00															
bio5	-0.78	-0.52	0.05	0.28	-0.11	0.08	-0.17	-0.12	-0.34	0.87	0.42	0.01	0.58	1.00														
bio6	-0.48	-0.22	0.03	0.21	0.01	0.07	-0.19	-0.09	-0.15	0.84	-0.24	0.00	-0.29	0.46	1.00													
bio7	-0.38	-0.34	0.03	0.11	-0.13	0.01	-0.01	-0.05	-0.23	0.16	0.64	0.01	0.86	0.63	-0.40	1.00												
bio8	-0.47	-0.38	-0.03	0.33	-0.02	0.12	-0.11	-0.07	-0.18	0.22	-0.10	-0.44	0.56	0.36	-0.01	0.37	1.00											
bio9	-0.57	-0.38	0.02	0.26	-0.11	0.09	-0.23	-0.12	-0.20	0.72	-0.07	-0.09	0.11	0.56	0.65	0.01	0.26	1.00										
bio10	-0.87	-0.56	0.04	0.33	-0.12	0.09	-0.22	-0.13	-0.38	0.91	0.15	-0.22	0.58	0.96	0.58	0.48	0.42	0.65	1.00									
bio11	-0.46	-0.22	0.04	0.20	0.00	0.06	-0.18	-0.10	-0.15	0.88	-0.02	0.19	-0.29	0.55	0.97	-0.29	-0.04	0.66	0.61	1.00								
bio12	0.76	0.50	-0.01	-0.30	0.03	-0.08	0.18	0.10	0.28	-0.45	0.22	0.63	-0.72	-0.57	-0.18	-0.43	-0.60	-0.48	-0.68	-0.12	1.00							
bio13	0.68	0.43	0.01	-0.31	-0.06	-0.09	0.17	0.09	0.21	-0.35	0.42	0.70	-0.55	-0.38	-0.22	-0.19	-0.54	-0.39	-0.53	-0.12	0.94	1.00						
bio14	0.05	-0.02	-0.03	0.19	-0.05	0.11	-0.12	0.06	-0.03	-0.11	-0.55	-0.43	-0.12	-0.28	0.09	-0.37	0.03	-0.13	-0.14	-0.03	0.10	-0.10	1.00					
bio15	0.45	0.26	0.00	-0.28	-0.01	-0.10	0.11	0.03	0.14	-0.19	0.42	0.60	-0.34	-0.20	-0.16	-0.06	-0.30	-0.08	-0.32	-0.06	0.66	0.81	-0.36	1.00				
bio16	0.67	0.42	0.00	-0.31	-0.05	-0.08	0.16	0.09	0.20	-0.35	0.38	0.67	-0.54	-0.39	-0.22	-0.21	-0.53	-0.39	-0.54	-0.13	0.94	0.99	-0.05	0.82	1.00			
bio17	0.49	0.34	-0.01	-0.10	0.03	0.00	0.06	0.09	0.15	-0.22	-0.14	0.23	-0.62	-0.44	0.09	-0.54	-0.40	-0.39	-0.45	0.08	0.76	0.61	0.58	0.18	0.62	1.00		
bio18	0.83	0.51	-0.03	-0.29	0.05	-0.08	0.21	0.10	0.33	-0.64	0.22	0.59	-0.64	-0.68	-0.40	-0.36	-0.47	-0.53	-0.81	-0.34	0.93	0.87	-0.01	0.69	0.88	0.58	1.00	
bio19	0.48	0.26	0.01	-0.14	-0.16	-0.01	0.07	0.09	0.07	-0.18	0.29	0.45	-0.35	-0.20	-0.11	-0.10	-0.42	-0.29	-0.30	-0.04	0.79	0.84	0.27	0.55	0.85	0.74	0.63	1.00
